# Supplementary material for: Axonal defasciculation is restricted to specific branching points during regeneration of the lateral line nerve in zebrafish
Source: bioRxiv. 2025 Jul 25:2025.07.23.666336. Preprint. [Version 1] doi: 10.1101/2025.07.23.666336 (PMC12330684; doi:10.1101/2025.07.23.666336)
Supplement: 1 [file NIHPP2025.07.23.666336V1-supplement-1.pdf]

bioRxiv preprint doi: https://doi.org/10.1101/2025.07.23.666336; this version posted July 25, 2025. The copyright holder for this preprint (which was not certified by peer review) is the author/funder, who has granted bioRxiv a license to display the preprint in perpetuity. It is made available under aCC-BY 4.0 International license.

Supplemental Information for:

Axonal defasciculation is restricted to specific branching points during regeneration of the lateral line nerve in zebrafish

Rohan S. Roy and A. J. Hudspeth

Laboratory of Sensory Neuroscience, The Rockefeller University, New York, NY 10065, USA

Howard Hughes Medical Institute, The Rockefeller University, New York, NY 10065, USA

bioRxiv preprint doi: https://doi.org/10.1101/2025.07.23.666336; this version posted July 25, 2025. The copyright holder for this preprint (which was not certified by peer review) is the author/funder, who has granted bioRxiv a license to display the preprint in perpetuity. It is made available under aCC-BY 4.0 International license.

Supplemental Information Titles

Supplemental Figure 1. Hair cells post nerve lesion

Supplemental Figure 2. FACS sorting of hair cells for bulk sequencing

Supplemental Figure 3. Distribution of p-values from bulk sequencing da

Supplemental Figure 4. Expression of axon guidance cues in denervated hair cells

Supplemental Figure 5. Normal nerve regeneration in $agrn^{p168}$ mutants

Supplemental Figure 6. Innervation of secondary neuromasts through collagen tunnel

Supplemental Figure 7. $col18a1a^{ru703}$ mutation

Supplemental Figure 8. Synaptic adhesion genes upregulated in denervated hair cells

Supplemental File 1. Spreadsheet of differential gene expression in hair cells one day post nerve lesion

Supplemental File 2. Spreadsheet of differential gene expression in hair cells three days post nerve lesion

bioRxiv preprint doi: https://doi.org/10.1101/2025.07.23.666336; this version posted July 25, 2025. The copyright holder for this preprint (which was not certified by peer review) is the author/funder, who has granted bioRxiv a license to display the preprint in perpetuity. It is made available under aCC-BY 4.0 International license.

Supplemental Figures and figure legends

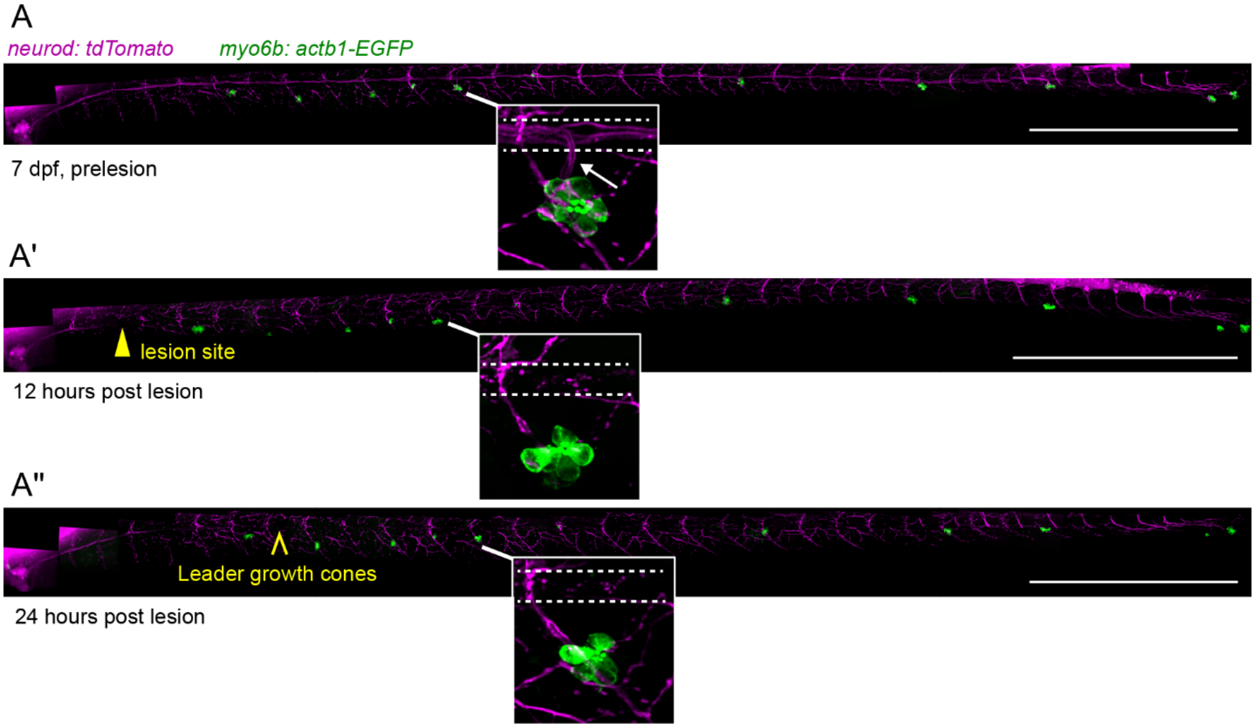

Supplemental Figure 1. Hair cells post nerve lesion.

(A) A representative 7 dpf *Tg(neurod:tdTomato, myo6b:actb1-EGFP)* larva before, 12 hpl (A'), and 24 hpl (A''). *Neurod* is a pan-neuronal promoter driving expression of tdTomato (magenta) and the *myo6b* promoter selectively labels hair cells with GFP (green). At 12 hpl, cut axons had not yet traversed the lesion site (yellow arrowhead). At 24 hpl, leader growth cones (yellow caret) had partially extended along the horizontal myoseptum. The majority of pLL hair cells were distal to the cut axons and remained denervated (inset). Dotted white lines demarcate the pLL nerve and the white arrow denotes axon defasciculation prior to lesion. Scale bar, 500 μm.

bioRxiv preprint doi: https://doi.org/10.1101/2025.07.23.666336; this version posted July 25, 2025. The copyright holder for this preprint (which was not certified by peer review) is the author/funder, who has granted bioRxiv a license to display the preprint in perpetuity. It is made available under aCC-BY 4.0 International license.

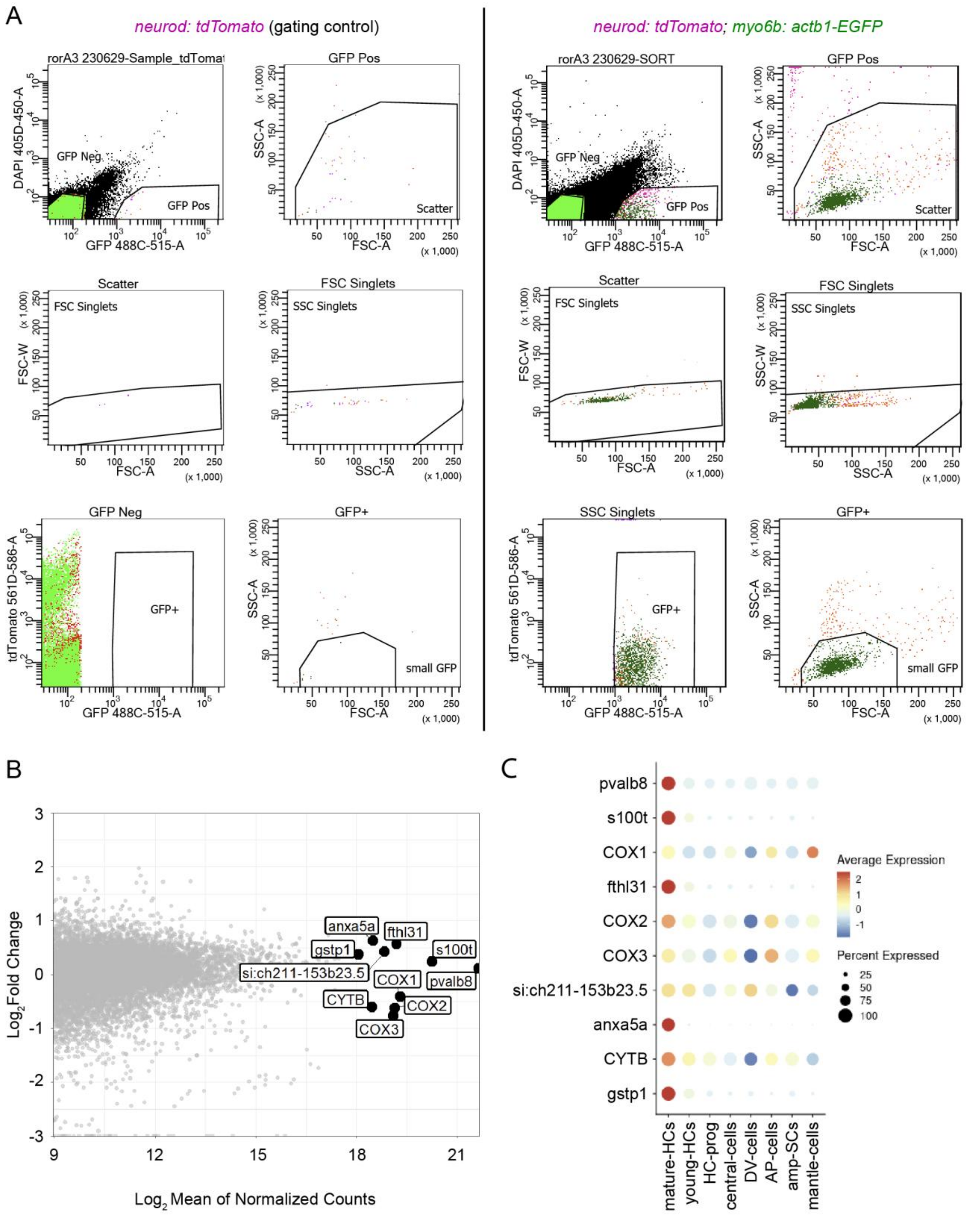

834 <u>Supplemental Figure 2. Isolation of hair cells for bulk sequencing</u>

bioRxiv preprint doi: https://doi.org/10.1101/2025.07.23.666336; this version posted July 25, 2025. The copyright holder for this preprint (which was not certified by peer review) is the author/funder, who has granted bioRxiv a license to display the preprint in perpetuity. It is made available under aCC-BY 4.0 International license.

(A) The gating strategy used for fluorescence-activated cell sorting (FACS) of GFP-positive hair cells in *Tg(neurod:tdTomato, myo6b:actb1-EGFP)* larvae following nerve lesion (right). Singly transgenic *Tg(neurod:tdTomato)* larvae were used as gating controls for each session (left). FSC – forward scatter, SSC – side scatter.

(B) An MA plot of transcripts sequenced in bulk hair cell samples 1 dpl compared to controls. The 10 most-expressed genes across all sample types are outlined in black.

(C) The same genes in (B) cross-referenced to a published single-cell RNAseq dataset of the neuromast (Baek et al., 2022). The genes are selectively and highly expressed in hair cells, confirming the accuracy of the isolation and sorting protocol.

bioRxiv preprint doi: https://doi.org/10.1101/2025.07.23.666336; this version posted July 25, 2025. The copyright holder for this preprint (which was not certified by peer review) is the author/funder, who has granted bioRxiv a license to display the preprint in perpetuity. It is made available under aCC-BY 4.0 International license.

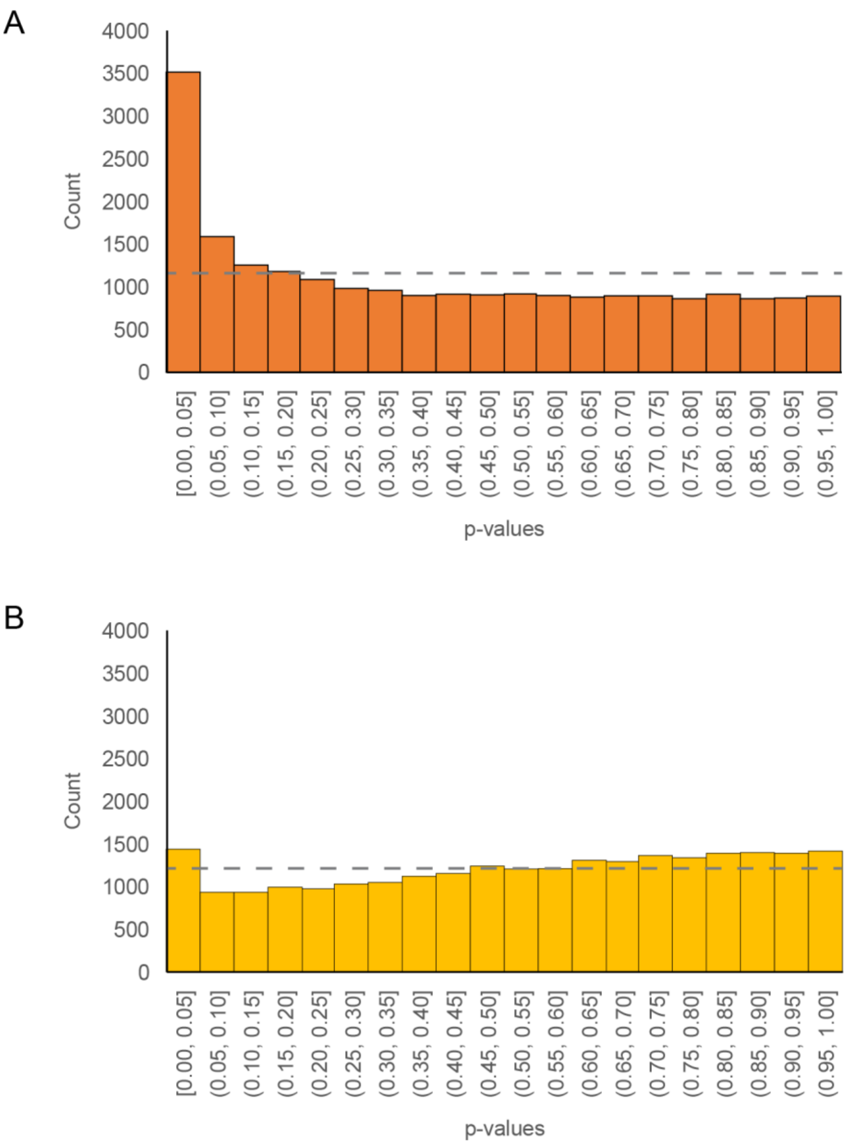

<u>Supplemental Figure 3. Distribution of p-values from bulk sequencing data</u>

(A) The distribution of *p-values* from testing the differential expression of each gene in hair cells 1 dpl compared to controls. A left-sided peak of values below 0.05 deviates from a uniform distribution (gray dotted line). n = 22,148 total genes tested.

(B) The distribution of *p-values* from testing the differential expression of each gene in hair cells 3 dpl compared to controls. The distribution more closely resembles a uniform distribution compared to (A). n = 24,209 total genes tested.

bioRxiv preprint doi: https://doi.org/10.1101/2025.07.23.666336; this version posted July 25, 2025. The copyright holder for this preprint (which was not certified by peer review) is the author/funder, who has granted bioRxiv a license to display the preprint in perpetuity. It is made available under aCC-BY 4.0 International license.

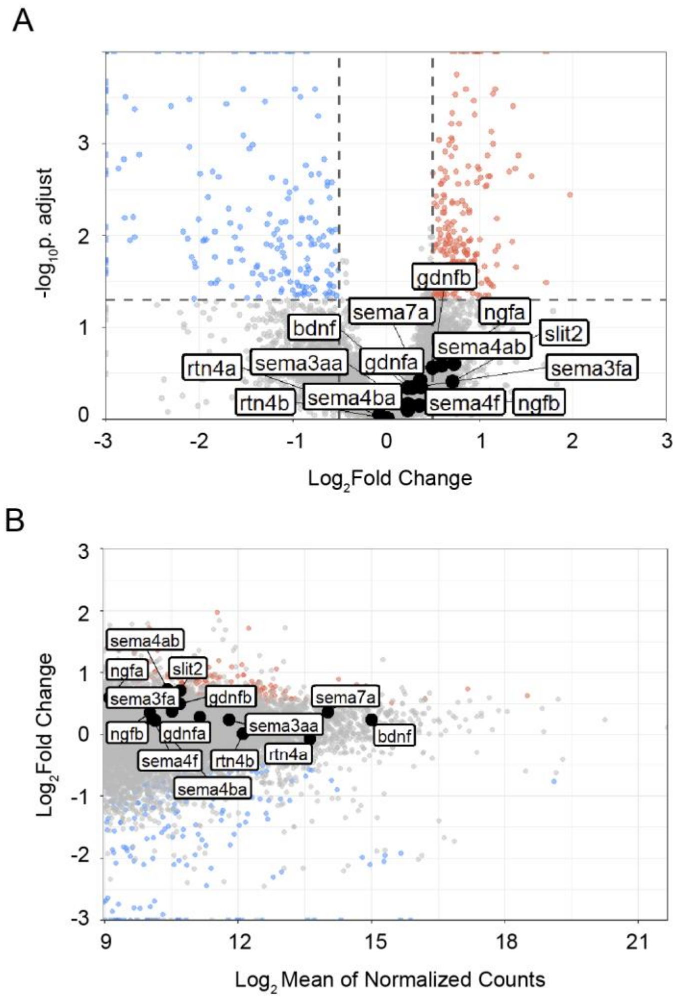

Supplemental Figure 4. Expression of axon guidance cues in denervated hair cells

(A) A volcano plot showing canonical axon guidance cues are not differentially expressed in hair cells 1 dpl compared to innervated controls. Statistical significance set at a *p-value* of 0.05 adjusted for multiple comparisons (horizontal dashed line) and biological significance set at 0.5 $\log_2$fold change (vertical dashed line).

(B) An MA plot of genes sequenced in hair cells 1 dpl compared to control with canonical axon guidance cues highlighted in black.

bioRxiv preprint doi: https://doi.org/10.1101/2025.07.23.666336; this version posted July 25, 2025. The copyright holder for this preprint (which was not certified by peer review) is the author/funder, who has granted bioRxiv a license to display the preprint in perpetuity. It is made available under aCC-BY 4.0 International license.

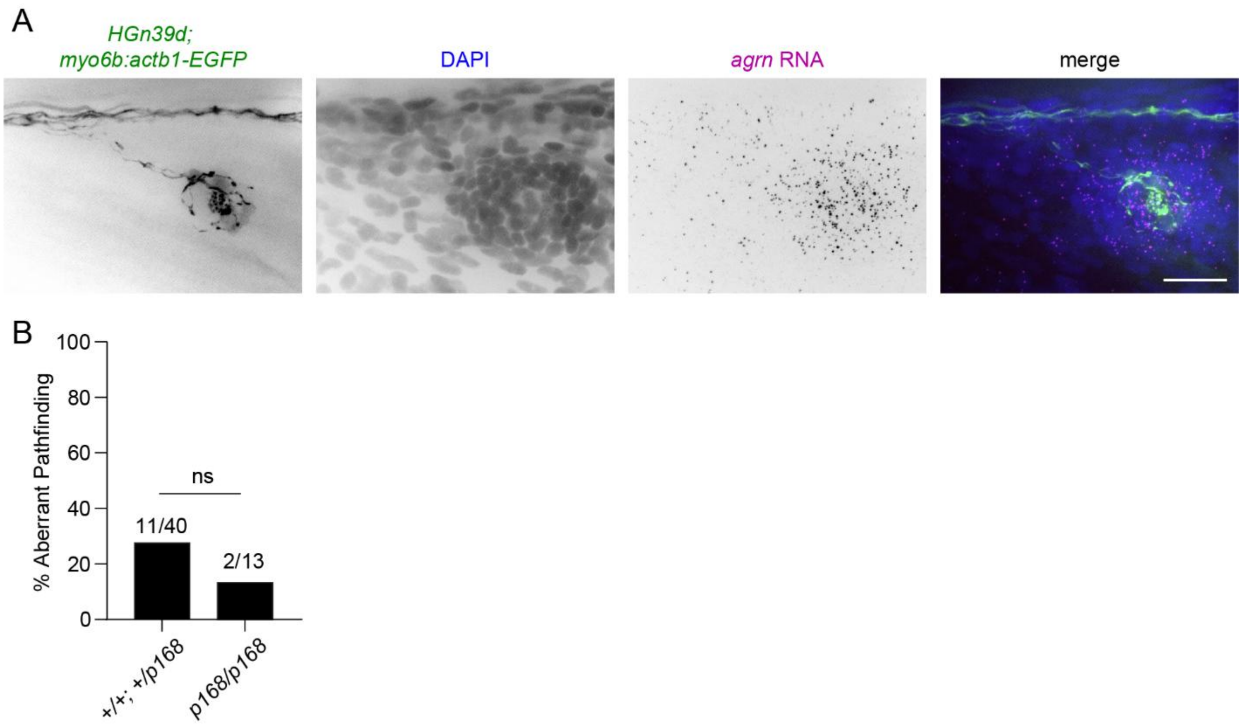

<u>Supplemental Figure 5. Normal nerve regeneration in *agrn*^{p168} mutants</u>

(A) RNA-FISH of *agrn* in a fixed 8 dpf *Tg(HGn39d; myo6b:actb1-EGFP)* larva. Expression of *agrn* is specific to hair cells and supporting cells of the neuromast.

(B) There is an absence of aberrant afferent axon pathfinding during nerve regeneration in *agrn*$^{p168/p168}$ *Tg(HGn39d; myo6b:actb1-EGFP)* mutants compared to siblings. n = 40 *+/+* and *+/p168* siblings, n = 13 *p168/p168* mutants. ns – not significant, Fisher's exact test.

bioRxiv preprint doi: https://doi.org/10.1101/2025.07.23.666336; this version posted July 25, 2025. The copyright holder for this preprint (which was not certified by peer review) is the author/funder, who has granted bioRxiv a license to display the preprint in perpetuity. It is made available under aCC-BY 4.0 International license.

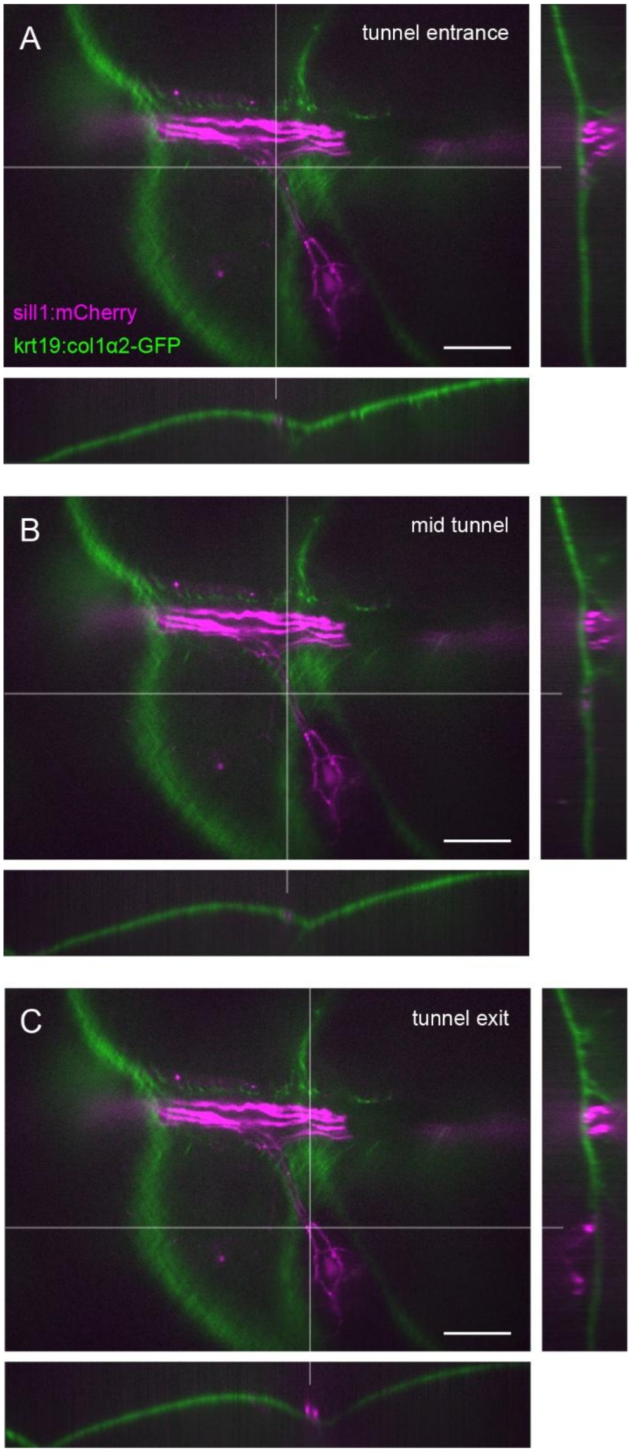

864 <u>Supplemental Figure 6. Innervation of secondary neuromasts through collagen tunnel</u>

865 (A) Orthogonal views of the point where individual axons defasciculate from the axon bundle to innervate a secondary neuromast in a 5 dpf *Tg(krt19:col1α2-GFP, sill1:mCherry)* larva.

bioRxiv preprint doi: https://doi.org/10.1101/2025.07.23.666336; this version posted July 25, 2025. The copyright holder for this preprint (which was not certified by peer review) is the author/funder, who has granted bioRxiv a license to display the preprint in perpetuity. It is made available under aCC-BY 4.0 International license.

The point of defasciculation coincides with the entrance to a tunnel within the collagen I matrix. Center – sagittal plane, right – axial plane, bottom – coronal plane.

(B) Orthogonal views of the midpoint of the collagen I matrix tunnel.

(C) Orthogonal views of the exit of the collagen I matrix tunnel at the secondary neuromast.

All scale bars, 20 μm.

bioRxiv preprint doi: https://doi.org/10.1101/2025.07.23.666336; this version posted July 25, 2025. The copyright holder for this preprint (which was not certified by peer review) is the author/funder, who has granted bioRxiv a license to display the preprint in perpetuity. It is made available under aCC-BY 4.0 International license.

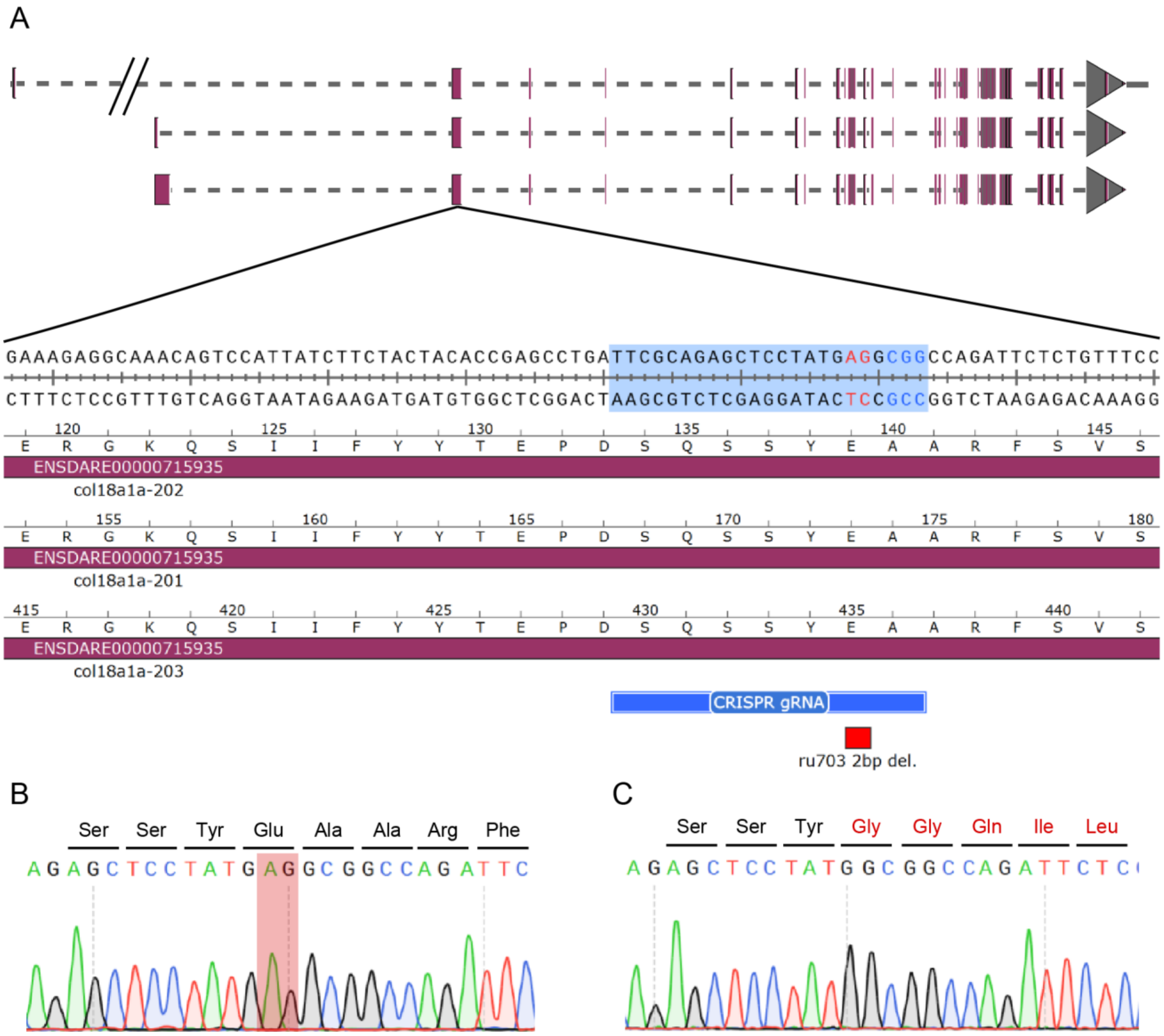

Supplemental Figure 7. *col18a1a*^{ru703} mutation

(A) The CRISPR gRNA sequence (highlighted) with adjacent CGG Cas9 PAM motif (blue) used to generate a stable *ru703* mutant line. The gRNA is complementary to either exon 2 in the 201 and 203 isoforms of *col18a1a*, and exon 3 in the 202 isoform. The two base pair AG deletion in *ru703* mutants are marked in red.

(B) A chromatogram of a sequenced wildtype larvae. AG base pairs deleted in the mutated *ru703* allele are highlighted in red.

bioRxiv preprint doi: https://doi.org/10.1101/2025.07.23.666336; this version posted July 25, 2025. The copyright holder for this preprint (which was not certified by peer review) is the author/funder, who has granted bioRxiv a license to display the preprint in perpetuity. It is made available under aCC-BY 4.0 International license.

(C) A chromatogram of a sequenced homozygous mutant *col18a1a*$^{ru703/ru703}$ larvae. The two base pair deletion creates a frameshift in the reading frame.

bioRxiv preprint doi: https://doi.org/10.1101/2025.07.23.666336; this version posted July 25, 2025. The copyright holder for this preprint (which was not certified by peer review) is the author/funder, who has granted bioRxiv a license to display the preprint in perpetuity. It is made available under aCC-BY 4.0 International license.

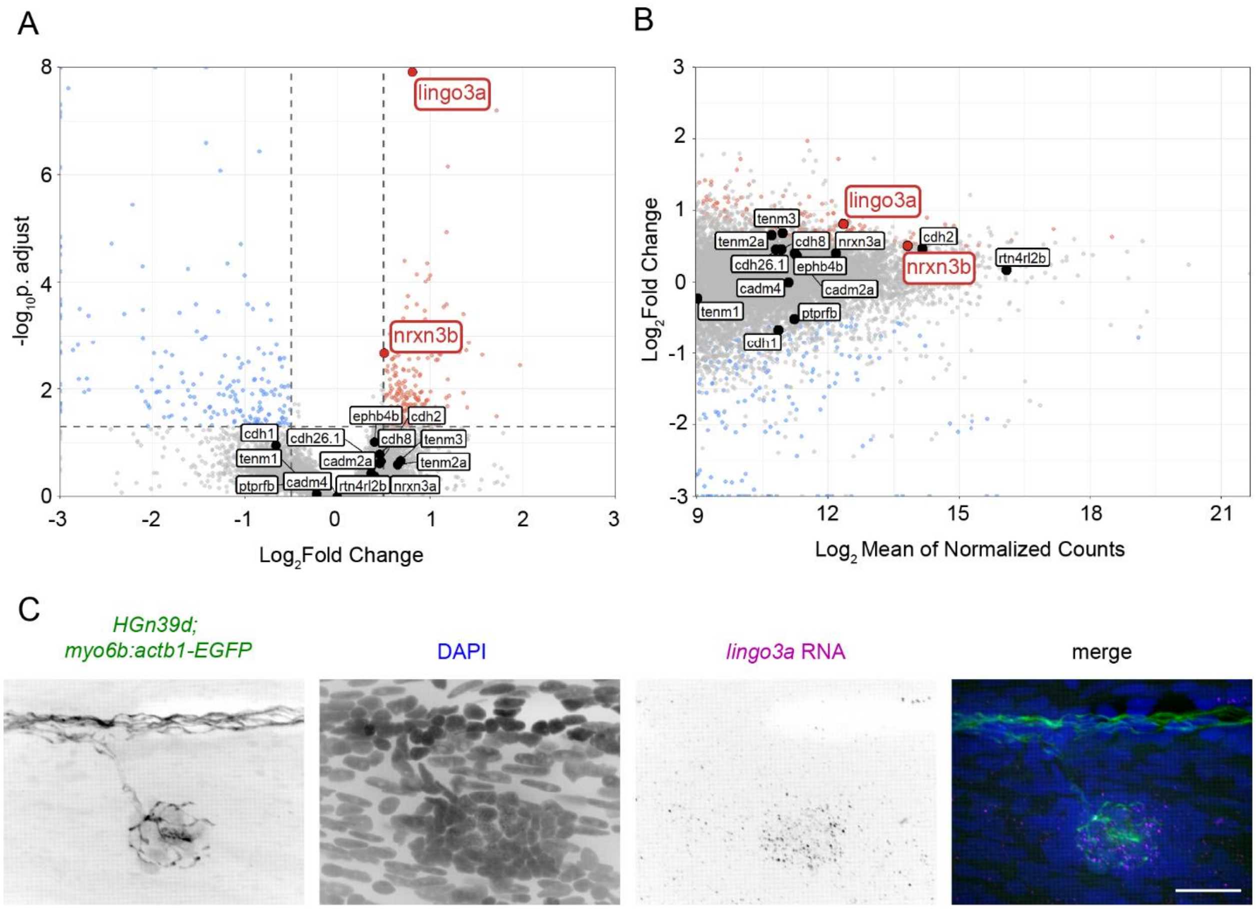

<u>Supplemental Figure 8. Synaptic adhesion genes upregulated in denervated hair cells</u>

(A) A volcano plot with canonical synaptic adhesion molecule genes highlighted. *Lingo3a* and *nrxn3b* are upregulated (red) in denervated hair cells 1 dpl compared to innervated controls. Statistical significance set at a p-value of 0.05 adjusted for multiple comparisons (horizontal dashed line) and biological significance set at 0.05 log_2fold change (vertical dashed line).

(B) An MA plot of genes sequenced in hair cells 1 dpl compared to control with canonical synaptic adhesion molecule genes highlighted.

(C) RNA-FISH against *lingo3a* in an 8 dpf *Tg(HGn39d; myo6b:actb1-EGFP)* larva reveals restricted expression in only hair cells of the neuromast. Scale bar, 20 µm.
